# Milk Containing A2 β-Casein ONLY, as a Single Meal, Causes Fewer Symptoms of Lactose Intolerance than Milk Containing A1 and A2 β-Caseins in Subjects with Lactose Maldigestion and Intolerance: A Randomized, Double-Blind, Crossover Trial

**DOI:** 10.3390/nu12123855

**Published:** 2020-12-17

**Authors:** Monica Ramakrishnan, Tracy K. Eaton, Omer M. Sermet, Dennis A. Savaiano

**Affiliations:** Department of Nutrition Science, College of Health and Human Sciences, Purdue University, West Lafayette, IN 47907, USA; ramakrm@purdue.edu (M.R.); tkeaton@purdue.edu (T.K.E.); omer_sermet@hotmail.com (O.M.S.)

**Keywords:** A1 beta-casein, A2 beta-casein, beta-casomorphin, gastrointestinal intolerance, hydrogen breath test, lactose challenge, lactose intolerance symptoms, milk intolerance, Qualifying Lactose Challenge Symptom Score

## Abstract

Acute-feeding and multiple-day studies have demonstrated that milk containing A2 β-casein only causes fewer symptoms of lactose intolerance (LI) than milk containing both A1 and A2 β-caseins. We conducted a single-meal study to evaluate the gastrointestinal (GI) tolerance of milk containing different concentrations of A1 and A2 β-casein proteins. This was a randomized, double-blind, crossover trial in 25 LI subjects with maldigestion and an additional eight lactose maldigesters who did not meet the QLCSS criteria. Subjects received each of four types of milk (milk containing A2 β-casein protein only, Jersey milk, conventional milk, and lactose-free milk) after overnight fasting. Symptoms of GI intolerance and breath hydrogen concentrations were analyzed for 6 h after ingestion of each type of milk. In an analysis of the 25 LI subjects, total symptom score for abdominal pain was lower following consumption of milk containing A2 β-casein only, compared with conventional milk (*p* = 0.004). Post hoc analysis with lactose maldigesters revealed statistically significantly improved symptom scores (*p* = 0.04) and lower hydrogen production (*p* = 0.04) following consumption of milk containing A2 β-casein only compared with conventional milk. Consumption of milk containing A2 β-casein only is associated with fewer GI symptoms than consumption of conventional milk in lactose maldigesters.

## 1. Introduction

Approximately 30% of cows’ milk protein is β-casein [1], of which two genetic variants exist: A1 and A2 [2]. A1 β-casein includes histidine at the 67th position in the peptide chain, whereas A2 β-casein includes proline at this position [3]. Although some cattle breeds maintain the A2 β-casein variant, a single nucleotide polymorphism in modern western cattle breeds means that they exhibit mixed A1 and A2 β-casein variants [3,4,5]. Digestive enzymes act on A1 β-casein and hydrolyze it, releasing beta-casomorphin-7 (BCM-7) [6,7,8,9,10]. The histidine residue in A1 β-casein allows cleavage to form BCM-7, whereas the proline residue in A2 β-casein limits such cleavage and BCM-7 formation [11].

In animal studies, BCM-7 is both pro-inflammatory and associated with slower gastrointestinal (GI) transit [12]. In intestinal and neuronal cells, BCM-7 downregulates the glutathione (GSH) levels [13], which is an important antioxidant in the body for combating oxidative stress, which otherwise can result in inflammation [14]. Oxidative stress has been shown to induce epigenetic changes, especially on genes that are important mediators of inflammation, leading to increased GI symptoms.

Multiple-day and acute-feeding studies in Chinese and Australian populations have shown that milk containing only the A2 β-casein protein caused fewer symptoms of lactose intolerance (LI) than milk containing both A1 and A2 β-casein protein [15,16,17,18]. However, these studies were conducted in subjects with self-reported LI, and no relevant blinded studies have been reported in verified LI individuals; this is a notable omission, given that self-reported LI can be unreliable [19]. Moreover, only one study to date has examined the effects of the administration of variable ratios of A1 and A2 β-casein [20]. Manifestations of LI can be both acute and chronic: they can be long-term due to epigenetic changes or genetic mutation [21]; they also appear to be a single-meal event, with symptoms occurring between 30 min and 6 h after exposure [22,23]. These symptoms and their cause are distinct from milk allergy, which results from an immune reaction to milk proteins.

We conducted a randomized, double-blind, single-meal feeding trial with four types of milk, which varied in A1/A2 β-casein protein ratio: milk containing A2 β-casein only, Jersey milk (containing 25%/75% A1/A2 β-casein), conventional milk (containing 75%/25% A1/A2 β-casein), and lactose-free milk. The principal study objective was to determine, via a hydrogen breath test (HBT) in lactose maldigester individuals living in the Midwest United States and who had verified LI following a blinded milk challenge, if lactose digestion and GI tolerance were affected by the four different milk types within 6 h after ingestion. We hypothesized that a single meal of milk containing A2 β-casein only would be better tolerated, producing fewer GI symptoms and less maldigestion during the 6 h study, than conventional milk containing both A1 and A2 β-casein. We also hypothesized that Jersey milk would produce an intermediate response regarding the HBT and occurrence of GI symptoms as a result of its higher level of A2 β-casein and lower level of A1 β-casein.

## 2. Materials and Methods

### 2.1. Subject Selection and Inclusion Criteria

This was a randomized, double-blind, crossover trial in subjects aged 18–65 years. Subjects were recruited through flyers and advertisements in local (West Lafayette, IN, USA) and campus (Purdue University, West Lafayette, IN, USA) newspapers, and Purdue Today email. Study recruitment started in February 2018 and was suspended in February 2020 due to COVID-19 restrictions by our institutional review board (IRB). A total of 853 people indicated interest in the study and contacted the investigators via email or phone. Of these, 258 subjects successfully completed phone screening and were then categorized as eligible or ineligible to participate in the trial (Appendix A). After the phone screens, all eligible subjects signed a consent form and agreed to participate in the study. We queried all interested subjects regarding demographic information, current medication use, and height and weight for calculating body mass index (BMI). Participants were assigned an identification number upon signing the informed consent form. Identification numbers were block pre-randomized using randomization.com. Staff performed the randomization, enrolled participants, and provided milk type information to our Clinical Research Center (CRC) kitchen via sealed security envelopes. Participants and study staff assessing outcomes over the 6 h were blinded to the milk types consumed.

Eligible subjects had to have avoided dairy for at least 1 month prior to screening and were included in the study only if they agreed to refrain from dairy and all treatments or products used for dairy intolerance (e.g., Lactaid^®^ dietary supplements; McNeil Nutritionals, LLC, Ft. Washington, PA, USA) throughout the trial. Subjects had to have a history of perceived dairy intolerance. Perceived LI was then confirmed by the Qualifying Lactose Challenge Symptom Score (QLCSS) during a 6 h HBT after consumption of a commercial milk containing a high amount of A1 β-casein [24]. Lactose maldigesters, defined as producing more than 20 parts per million (ppm) hydrogen at any time point following the baseline commercial milk challenge [25], were eligible for the intervention portion of the study.

Abdominal pain, bloating, flatulence, and diarrhea are typical symptoms of LI [26]. Although other studies of A1 and A2 β-caseins reported scores for stool frequency and stool consistency as indicators of LI [15,16,17], in the current study, qualifying scores for abdominal pain, bloating, flatulence, diarrhea, and fecal urgency were each recorded by subjects using a six-point Likert scale. Symptoms were ranked from 0 to 5, where 0 was for no symptoms, 1 for slight, 2 for mild, 3 for moderate, 4 for moderately severe, and 5 for severe symptoms. If subjects met one of the following three criteria regarding the QLCSS, they were classified as lactose intolerant: a score of 4 or 5 for an individual symptom; a score of 3 for at least two symptoms; or a score of 3 for one symptom at two time points in the study.

### 2.2. Exclusion Criteria

Subjects were excluded from the study for the following reasons: allergy to milk; pregnancy or lactation; cigarette smoking, or use of tobacco or nicotine-containing products within 3 months of screening; diagnosis of abnormal GI motility; a history of GI tract surgery; the presence of any medical condition with symptoms that could confound collection of data about adverse events; ulcer; diabetes mellitus; congestive heart failure; HIV, hepatitis B, or hepatitis C virus infection; BMI > 35 kg/m^2^; use of products to treat dairy intolerance within 7 days of screening; use of antacids and/or proton pump inhibitors; use of antibiotics or colonic enemas within 30 days prior to screening; any concurrent disease or symptoms that may interfere with assessment of the cardinal symptoms of dairy intolerance; use of ethanol (alcohol) and/or drug abuse in the past month; chemotherapy; or use of any investigational drug, or participation in any investigational study, within 30 days prior to screening.

### 2.3. Interventions

Four types of milk were evaluated in the study: milk containing A2 β-casein only (The a2 Milk Company, Boulder, CO, USA); Jersey milk (containing 25%/75% A1/A2 β-casein; American Jersey Cattle Association, Crockett, VA, USA); conventional milk (containing 75%/25% A1/A2 β-casein; Kroger^®^ 2% reduced fat; The Kroger Co., Indianapolis, IN, USA); and lactose-free milk (containing 60%/40% A1/A2 β-casein; Lactaid^®^; McNeil Nutritionals, LLC). Jersey milk was shipped to our study laboratory (Purdue University, West Lafayette, IN, USA) from the American Jersey Cattle Association (Crockett, VA, USA). All other milk products were purchased from Payless (West Lafayette, IN, USA); if they were unavailable at Payless, then they were purchased from Fresh Thyme (West Lafayette, IN, USA). All four milk types were procured every two weeks and administered prior to their expiry dates.

### 2.4. A1/A2 Analysis

The ratio of A1/A2 β-casein in the four types of milk was analyzed using mass spectrometry (MS) at Purdue Proteomics Facility. Protein extraction from 100 µL of each milk was performed by denaturation with 400 µL of 8 M urea and 10 mM dithiothreitol (DTT) and vortexing for 15 min at room temperature to remove fat. This was followed by protein alkylation with 400 µL of 8 M urea and 10 mM DTT, and then digestion with pepsin using a 1:20 enzyme to substrate ratio for 1 h at room temperature. The peptides, which were cleaned/desalted via a C18 Silica MicroSpin column (The Nest Group Inc., Southborough, MA, USA) after digestion, were analyzed with the Dionex UltiMate 3000 RSLC nano System combined with the Q-Exactive High-Field Hybrid Quadrupole Orbitrap MS (Thermo Fisher Scientific, Waltham, MA, USA). Peptides were then re-suspended in 3% acetonitrile/0.1% formic acid/96.9% Milli-Q water, and 5 μL (1 μg) were used for liquid chromatography (LC)-MS/MS analysis. A trap (300 μm internal diameter (ID) × 5 mm packed with 5 μm 100 Å PepMap C18 medium; Thermo Fisher Scientific) was used to separate peptides and a 120 min gradient method with a flow rate of 300 nL/min was used for the analytical columns (75 μm ID × 15 cm long packed with 3 μm of 100 Å PepMap C18 medium). Mobile phase A contained 0.1% formic acid in water and mobile phase B contained 0.1% formic acid in 80% acetonitrile. The linear gradient started at 5% B and reached 30% B in 80 min, 45% B in 91 min, and 100% B in 93 min. The column was held at 100% B for 5 min and then brought back to 5% B. The column was held at 5% B for 20 min to equilibrate at 37 °C. The top 20 data-dependent MS/MS scan method was used to acquire MS data with a maximum injection time of 100 ms and a resolution of 120,000 at 200 *m*/*z*. High-energy C-trap dissociation with the normalized collision energy of 27 eV was used to fragment precursor ions. MS/MS scans were acquired at a resolution of 15,000 at 200 *m*/*z*. Repeated scanning of identical peptides was avoided by setting the dynamic exclusion at 20 s.

LC-MS/MS data were analyzed using MaxQuant software (version 1.6.0.1; Max Planck Institute of Biochemistry, Martinsried, Germany). The combined non-redundant *Bos taurus* protein sequence database downloaded from UniProt (www.uniprot.org) in January 2017 was used for protein identification and label-free relative quantitation. The following parameters were used for database searches: precursor mass tolerance of 10 ppm; enzyme pepsin allowing up to two missed cleavages; oxidation of methionine as a variable modification and iodoethanol as a fixed modification. The false discovery rate of peptide spectral match and protein identification was set to 0.01. Only proteins with a label-free quantitation value of 0 and MS/MS spectral counts of ≥2 were considered as a true identification before being used for further analysis. 

### 2.5. Sugar, Protein, and Fat Analyses

Total sugars, fat, and protein were analyzed by Eurofins Food Integrity and Innovation (Eurofins Food Chemistry Testing US, Inc., Madison, WI, USA) [27,28,29]. The sugar profile was determined using 10 g of each milk type (with the exception of lactose-free milk), and sugars were extracted with a 50:50 methanol:water solution. Inert gas was used to dry each sample, which was derivatized prior to analysis, and the analysis was conducted via gas chromatography with flame ionization detection.

Because of its very low lactose content, lactose-free milk was analyzed using a different procedure. A 10 g milk sample was extracted with dilute HCl and centrifuged. The supernatant was filtered using a strong cation exchange cartridge, and an OnGuard II syringe filter (Thermo Fisher Scientific) was used for neutralization. Applicable amounts of dilutions were injected into a high-performance anion exchange chromatography system equipped with pulsed amperometric detection (Thermo Fisher Scientific).

Fat in the samples was analyzed by base hydrolysis, and protein was analyzed using the Dumas method [27].

### 2.6. Study Procedures

The subjects reported to our clinical research facility (Purdue University) for four visits, with at least six days between any two consecutive visits. Subjects consumed a low-fiber dinner and fasted for 12 h prior to visits. Subjects consumed a different randomized milk product at 8 a.m. on the day of each visit. Each milk meal, except the lactose-free milk, contained ~4.5 g of lactose/per 100 mL. The amount of milk consumed was calculated as approximately 0.5 g of lactose times bodyweight in kg, divided by 11 g (the normal amount of lactose in a cup of regular milk), then multiplied by 245 mL (one cup).

$$\mathrm{Amount}\;\mathrm{of}\;\mathrm{milk}\;\mathrm{consumed}=\frac{0.5\;g\;\mathrm{of}\;\mathrm{lactose}\times \mathrm{bodyweight}\;\left(\mathrm{kg}\right)}{11\;g\;\mathrm{of}\;\mathrm{lactose}}\times 245\;\mathrm{mL}\;\mathrm{of}\;\mathrm{milk}$$

Subjects always consumed the same quantity of fluid (mL) and lactose (g), despite small variations in the lactose content.

### 2.7. Study Endpoints

The primary study endpoints were the occurrence of GI symptoms (abdominal pain, bloating, flatulence, diarrhea, and fecal urgency), and measurement of hydrogen in breath samples (a standard measure of maldigestion), for up to 6 h after consumption of each milk type. Breath samples were collected, and GI symptoms were recorded by the subjects, at 0, 0.5, 1, 1.5, 2, 3, 4, 5, and 6 h after commercial milk ingestion. To measure hydrogen in the breath samples, a hydrogen microanalyzer (QuinTron BreathTracker Digital Microlyzer, model SC; QuinTron Instrument Company, Inc., Milwaukee, WI, USA) was used. An increase of 20 ppm hydrogen between any two timepoints in the study indicated lactose maldigestion [26]. Symptoms were scored using a six-point Likert scale (as described in the inclusion criteria).

### 2.8. Study Ethics

The study (ClinicalTrials.gov #NCT03713346) and its protocol were approved by the Purdue IRB (IRB #1710019781). The trial was conducted in accordance with the Helsinki Declaration of 1975 as revised in 1983. The study was also conducted in accordance with International Conference on Harmonization Good Clinical Practice guidelines.

### 2.9. Statistical Analyses

The initial power calculation indicated a sample size requirement of 26, which was determined based on the selection of a 20% decrease in area under the curve for change in breath hydrogen as the minimal difference that would be clinically significant. Power calculations indicated that completion of the protocol, with a crossover study design, by 26 subjects would be adequate to demonstrate 80% statistical power, consistent with biological relevance using α = 0.05 to detect a 20% change in breath hydrogen. The sample size for symptoms was derived from a previous study in Chinese preschoolers aged 5 to 6 years [18]. The COVID-19 pandemic caused us to suspend the study in February 2020 with 25 verified LI subjects and an additional eight maldigesters who did not meet the LI criteria. However, we observed that not all LI subjects met the criteria for LI after a second commercial milk dose, suggesting that the symptom criteria were arbitrary and inconsistent. A post hoc analysis indicated only 15 of 25 LI subjects met the LI criteria after receiving the second commercial milk challenge as part of the randomized intervention; therefore, we included maldigesters who did not meet the criteria for LI in the study in June 2019, to better understand the potential effect of milk containing A2 β-casein only and Jersey milk on all maldigesters. As a result, an analysis was conducted with 25 LI subjects, and a post hoc analysis was conducted with the addition of eight maldigesters (a total of 33 subjects).

GI symptoms, hydrogen at each timepoint, and total hydrogen during each 6 h study period were analyzed using the paired *t*-test. Two-tailed *p*-values were compared with a significance level of 0.05. Descriptive statistics were used to calculate mean and standard error values for GI symptoms and breath hydrogen. All statistical analyses were conducted using Microsoft^®^ Excel and the Statistical Package for the Social Sciences (IBM SPSS Statistics for Windows, Version 26.0; IBM Corp., Armonk, NY, USA).

Lactose-free milk was used as a negative control. Conventional milk was compared with Jersey milk and milk containing A2 β-casein only, by measuring the occurrence of GI symptoms and breath hydrogen. Subject symptom scores for abdominal pain, bloating, flatulence, and diarrhea were summed over 6 h after each milk consumption, and total symptoms for each subject were calculated as the sum of all the total symptom scores for abdominal pain, bloating, flatulence, and diarrhea; symptoms of fecal urgency were analyzed separately. For each subject, baseline breath hydrogen concentration was subtracted from the breath hydrogen concentration produced at each timepoint (0, 0.5, 1, 1.5, 2, 3, 4, 5, and 6 h) to correct for residual hydrogen.

## 3. Results

### 3.1. Baseline and Demographic Characteristics

**Figure 1:**
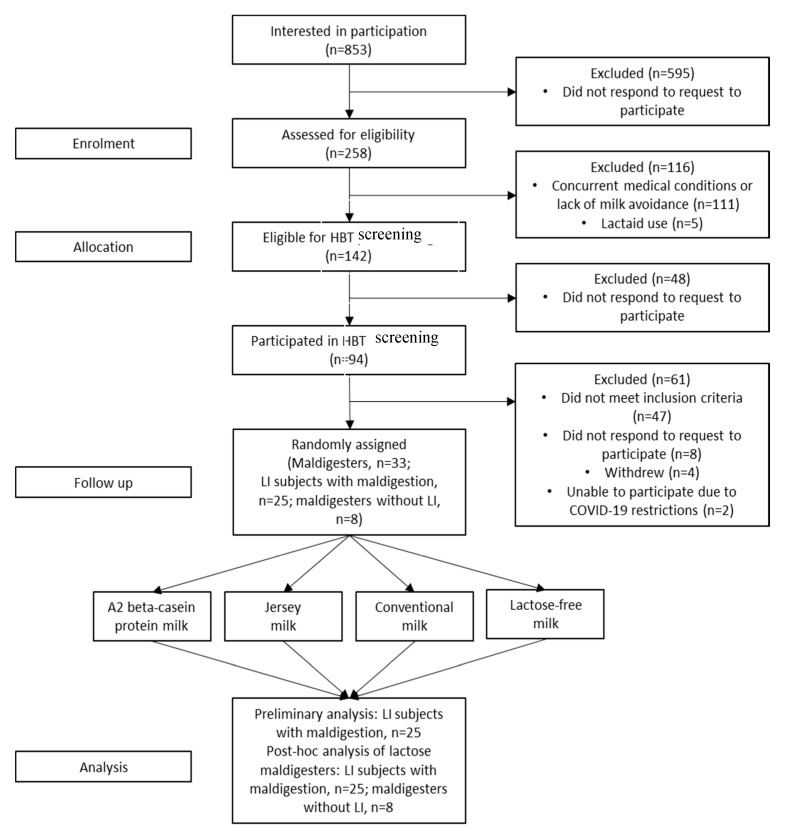
Study enrollment, randomization and analyses. HBT, hydrogen breath test; LI, lactose intolerance/intolerant.

Of the 258 subjects who were phone-screened, 111 were ineligible due to medical conditions or lack of milk avoidance, and five subjects were ineligible because of Lactaid^®^ use. A total of 142 subjects were eligible for the HBT screening, but only 94 chose to participate; the other 48 subjects did not respond to attempts to schedule the baseline screening. A total of 35 subjects met the maldigestion criteria and were randomized to one of four sequences for receiving the four milk products. Thirty-three completed the four study visits and had GI symptoms and hydrogen production in HBTs recorded for 6 h after each milk treatment. Of the 33 subjects, 25 met the symptom criteria and were classified as LI and an additional eight subjects were maldigesters without LI; two subjects were unable to complete the protocol owing to COVID-19 restrictions implemented by our IRB (Figure 1).

Overall, 15 male and 18 female subjects with a mean age of 25 (range 19–50) years, and a mean BMI of 24 (range 18–33) kg/m^2^, completed the study. The study population comprised 14 individuals who identified as Asian, four African Americans, 14 Caucasians, and one American Indian. All participants resided in the United States: five were Hispanic, 26 were non-Hispanic, and two participants did not disclose ethnicity (Hispanic/non-Hispanic) (Table 1). Nutrient composition for each of the four milk products evaluated is shown in Table 2.

### 3.2. GI Symptoms

#### 3.2.1. LI Subjects

**Figure 2:**
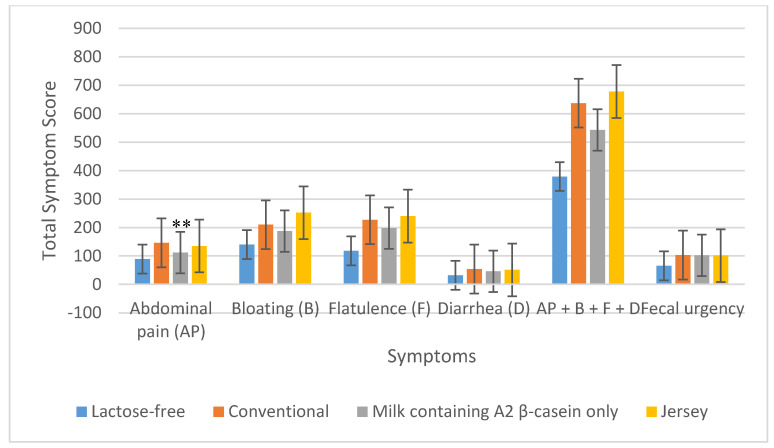
Total symptoms reported during the 6 h after consuming the four milk products in 25 lactose intolerant subjects. ** *p* = 0.004 for abdominal pain due to milk containing A2 β-casein only vs. conventional milk.

The total symptom score for abdominal pain during the 6 h after the consumption of milk containing A2 β-casein only, in LI subjects (*n* = 25), was significantly lower than that following consumption of conventional milk (112 vs. 146; *p* = 0.004); however, in contrast, the total score for abdominal pain after consumption of Jersey milk was not significantly different from that for conventional milk (135 vs. 146; *p* = 0.63). Regarding total symptom scores for bloating, flatulence, diarrhea, and fecal urgency, no significant differences were evident between those who had consumed conventional milk and those who had consumed either Jersey milk or milk containing A2 β-casein only (Figure 2). With respect to the combined total symptom scores for abdominal pain, bloating, flatulence, and diarrhea reported by subjects, there were no significant differences between conventional milk and Jersey milk or milk containing A2 β-casein only (Table 3 and Figure 2).

#### 3.2.2. All Maldigesters

**Figure 3:**
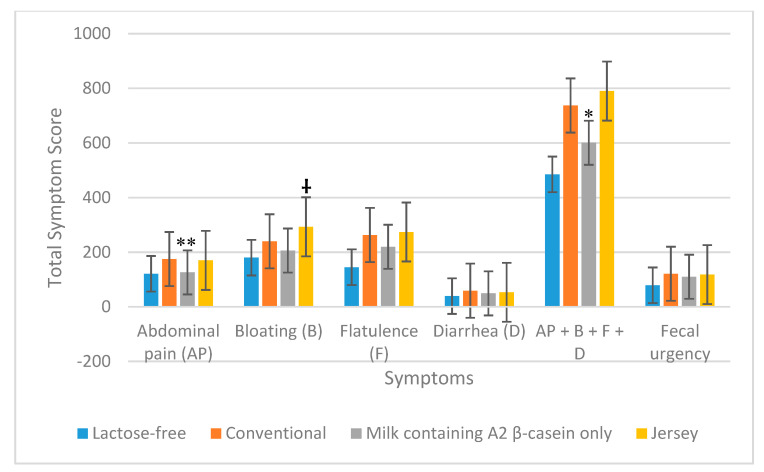
Total symptoms reported during the 6 h after consuming the four milk products in 33 lactose maldigesters. ** *p* = 0.001 for abdominal pain and * *p* = 0.04 for total symptoms (abdominal pain + bloating + flatulence + diarrhea) due to milk containing A2 β-casein only vs. conventional milk; † *p* = 0.05 for bloating due to Jersey milk versus conventional milk.

The total symptom score for abdominal pain after the consumption of milk containing A2 β-casein only, in all maldigesters (*n* = 33), was significantly lower than that following consumption of conventional milk (126 vs. 175; *p* = 0.001); however, the total score for abdominal pain after the consumption of Jersey milk was not significantly different from that for conventional milk (170 vs. 175; *p* = 0.83). Total symptom score for bloating was higher when consuming Jersey milk compared with conventional milk (293 vs. 240; *p* = 0.05). Total symptom scores for flatulence, diarrhea, and fecal urgency were similar in subjects consuming milk containing A2 β-casein only, Jersey milk, and conventional milk (Figure 3). The combined total symptom scores for abdominal pain, bloating, flatulence, and diarrhea showed there were fewer symptoms with milk containing A2 β-casein only (601 vs. 737; *p* = 0.04) compared with conventional milk, whereas consumption of Jersey milk or conventional milk produced similar symptom scores (790 vs. 737; *p* = 0.44) (Table 3 and Figure 3).

### 3.3. HBT Results

#### 3.3.1. LI Subjects

**Figure 4:**
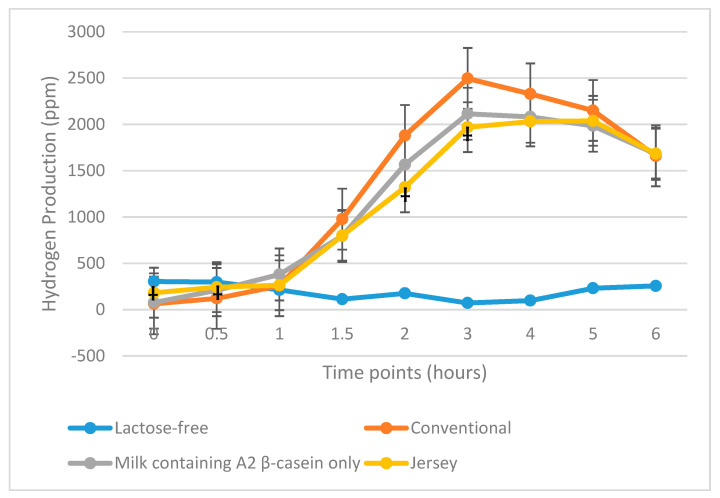
Total hydrogen produced during the 6 h after consuming the four milk products in 25 lactose intolerant subjects. ppm, parts per million. † *p* = 0.05, † *p* = 0.03, † *p* = 0.01, and † *p* = 0.05 for Jersey milk vs. commercial milk at 0, 0.5, 2, and 3 h, respectively.

Hydrogen breath concentration was analyzed in 25 LI subjects. The total quantity of hydrogen produced was not significantly different during the 6 h after the consumption of Jersey milk or milk containing A2 β-casein only when compared with consumption of conventional milk (Table 3 and Figure 4).

#### 3.3.2. All Maldigesters

**Figure 5:**
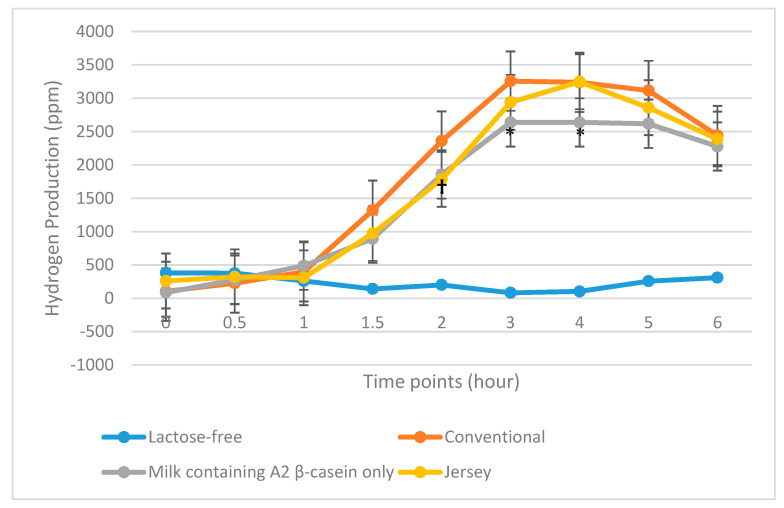
Total hydrogen produced during the 6 h after consuming the four milk products in 33 lactose maldigesters. ppm, parts per million. * *p* = 0.05, * *p* = 0.03 for milk containing A2 β-casein only vs. conventional milk at 3 and 4 h, respectively; † *p* = 0.03 for Jersey milk versus conventional milk at 2 h.

Total hydrogen produced by 33 maldigesters following consumption of milk containing A2 β-casein only was significantly lower compared with hydrogen produced by subjects following consumption of conventional milk (13,771 vs. 16,460 ppm; *p* = 0.04). However, hydrogen production following consumption of Jersey milk was not significantly different from that following consumption of conventional milk (15,079 vs. 16,460 ppm; *p* = 0.17) (Table 3 and Figure 5).

### 3.4. Adverse Events

There were no adverse events or unintended harmful effects reported by subjects due to the consumption of the four different types of milk.

## 4. Discussion

The results of our study indicate that the consumption of milk containing A2 β-casein only produced fewer GI symptoms in lactose maldigesters compared with consumption of conventional milk. On the other hand, Jersey milk did not reduce GI symptoms, compared with conventional milk. In LI subjects and lactose maldigesters, milk containing only A2 β-casein significantly decreased abdominal pain compared with the consumption of conventional milk. Conversely, the consumption of Jersey milk was not associated with reduced abdominal pain.

The effects of the milk treatments on GI symptoms may be related to GI effects due to longer transit time in the colon by milk containing A1 β-casein [17]. A study in Wistar rats and some human clinical trials showed that A1 β-casein increased GI transit time and colonic activity of the inflammatory marker myeloperoxidase [17,18,30]. These effects, which were counteracted by the opioid blocker naloxone, might be initiated and mediated by the opioid peptide BCM-7, which is formed after the ingestion of A1 β-casein [30]. Further, bovine casein-derived opioid peptides can inhibit cysteine uptake in both GI epithelial and neuronal cells, resulting in elevated oxidative stress and altered DNA methylation, including on genes that are important for mediating inflammation [31].

Consistent with the results reported herein, in Chinese subjects with self-reported LI, consumption of conventional milk (equivalent to the conventional milk tested in the present study) produced more GI symptoms than did milk containing A2 β-casein only [17]. Moreover, increased GI transit times and concentrations of serum inflammatory markers IgG and IL-4 were noted after the consumption of milk containing A1/A2 β-casein rather than milk containing A2 β-casein only. Therefore, the increase in GI symptoms may be due to inflammation and GI transit time, suggesting a need for further investigation.

The presence of LI should be confirmed by recording symptom scores for abdominal pain, bloating, flatulence, and diarrhea [26], and previous studies of A1 and A2 β-caseins in LI individuals did not specifically use QLCSS to screen subjects [15,16,17,18]; as a result, these earlier studies selected subjects with perceived LI for evaluation, many of whom may not have been truly intolerant. In contrast, we verified LI via symptom scores during screening. Thus, our study is the first to demonstrate that verified LI individuals are able to better tolerate a single meal of milk containing only A2 β-casein compared with conventional milk containing both A1 and A2 β-casein.

Because our study included only 25 LI subjects, the results may not be generalizable to larger populations, although this limitation is offset by the racial and ethnic diversity of the study population. Notably, the strict QLCSS inclusion criteria contributed to the small sample size. However, given the greater statistical significance when including the eight maldigesters without symptoms of intolerance, this rigorous inclusion criterion might not be important in the population we studied. Furthermore, the fact that only 35 of 94 eligible milk avoiders/perceived intolerant individuals met the maldigestion criteria suggests that the number of people in our population with perceived or self-reported LI markedly exceeds the number with actual LI verified by symptom scores and maldigestion.

We did not categorize the subjects into age groups. Among the 25 LI study participants, 22 were in the 19- to 35-year-old age group, and three were in the 36- to 50-year-old age group. However, in a study of 600 participants aged 20–50 years who were stratified into two groups (20–35 years and 36–50 years), age had no effect on GI symptoms after milk consumption [15]; nonetheless, adults aged >50 years might respond differently to milk ingestion and this requires further evaluation.

The BMI of subjects in our study ranged from 18–33 kg/m^2^. Normal-weight, overweight, and obese subjects were included in the study, but severely obese individuals were excluded. The impact of BMI differences on GI symptoms was reduced by providing subjects with calculated quantities of milk with respect to bodyweight, something that was not done in previous studies [15,16,17,18].

The effects of A1 β-casein, or its digestive by-product BCM-7, appear to be acute in our study. That is, the effects of a single milk challenge in our study were monitored over a short period (30 min to 6 h), and results may have been different if a multi-meal or multi-day feeding trial had been conducted in the same population. Long-term feeding effects might worsen GI symptoms and prior studies have shown sustained inflammatory effects with A1 β-casein, which could worsen symptoms [17,18,32]. Furthermore, there are also changes in microbial metabolites such as butanoic acid, acetic acid, and propanoic acid in adults and children following consumption of A1 β-casein [17,18], showing that A1 β-casein affects the microbiota in the gut.

## 5. Conclusions

In summary, results of the analysis of 25 LI subjects revealed significantly lower abdominal pain after the consumption of milk containing only A2 β-casein compared with the consumption of conventional milk. Total breath hydrogen produced by LI subjects was not significantly different from that after the consumption of conventional milk, possibly because our sample size was too small to detect differences in breath hydrogen production. The reduction in abdominal pain after the consumption of milk containing A2 β-casein only, compared with the consumption of conventional milk, was consistent with the results of another clinical trial [15]. Among the eight maldigesters tested since June 2019, none met the LI criteria during screening or intervention. In the post hoc analysis, symptoms of intolerance were not reduced after the consumption of Jersey milk compared with conventional milk, potentially because of the presence of some A1 β-casein in Jersey milk. However, there was a significant reduction in symptoms among these 25 individuals and eight additional lactose maldigesters following the consumption of milk containing only A2 β-casein. These findings warrant confirmation in larger study populations.

## Figures and Tables

**Table 1:** Baseline and demographic characteristics.

Age, mean (range); years	25 (19–50)
Bodyweight, mean (range); kg	71
Height, mean (range); cm	170
BMI, mean (range); kg/m^2^	24
Male/female, *n*/*n*	15/18
Lactose intolerant maldigesters (meeting QLCSS; hydrogen > 20 ppm), *n*	25
Lactose tolerant maldigesters (hydrogen ≤ 20 ppm), *n*	8
Race, *n*	
Asian	14
African American	4
Caucasian	14
American Indian	1
Ethnicity, *n*	
Hispanic	5
Non-Hispanic	26
Unknown	2

Baseline and demographic characteristics of maldigesters and lactose intolerant subjects (*n* = 33) enrolled in this randomized, double-blinded trial comparing conventional milk with Jersey milk and milk containing A2 β-casein only. BMI, body mass index; ppm, parts per million; QLCSS, Qualifying Lactose Challenge Symptom Score.

**Table 2:** Nutrient composition of the four milk treatments.

Nutrient	Milk Containing A2 β-Casein Only	Jersey Milk	Conventional Milk	Lactose-Free Milk
Protein (g/serving)	3.14	3.95	3.30	3.21
Fat (g/serving)	2.10	2.00	1.90	2.00
Lactose (g/serving)	4.70	4.40	4.60	0.13
Carbohydrate (g/serving)	4.70	4.40	4.60	*N*/A
Calories (kcal/serving)	0.0541	0.0500	0.0500	0.0500
A1 β-casein protein (%)	0.00	25.00	75.00	60.00
A2 β-casein protein (%)	100.00	75.00	25.00	40.00

Subjects were fed approximately 4.5 g lactose/100 mL of each milk after an overnight fast, in random order, with six days between treatments.

**Table 3:** Comparison of total hydrogen produced, and symptoms reported.

Criteria	Pairs	LI Subjects (*n* = 25)		Lactose Maldigesters (*n* = 33; 25 LI Subjects + 8 Maldigesters)	
		Total	*p*-Values	Total	*p*-Values
Total hydrogen produced per subject (ppm)	Conventional milk Milk containing A2 β-casein only	11,935 10,892	0.31	16,460 13,771	0.04
	Conventional milk Jersey milk	11,935 10,533	0.09	16,460 15,079	0.44
Total symptom scores ^a^	Conventional milk Milk containing A2 β-casein only	637 543	0.13	737 601	0.04
	Conventional milk Jersey milk	637 678	0.55	737 790	0.17

Comparison of total hydrogen produced, and symptoms reported for six hours following consumption of conventional milk versus milk containing A2 β-casein only and conventional milk versus Jersey milk using paired *t*-tests. LI, lactose intolerant; ppm, parts per million; ^a^ abdominal pain + bloating + flatulence + diarrhea.

## Appendix A

### Inclusion and Exclusion Criteria for Phone Screening

Inclusion criteria:

1. Ability/desire to provide informed consent
2. Aged 18–65 years at screening
3. Current or recent history of intolerance to and avoidance of dairy for at least 1 mo (by self-report and self-reported symptoms).
4. Agreement to refrain from all other treatments and products used for dairy intolerance (e.g., Lactaid^®^ dietary supplements; McNeil Nutritionals, LLC, Ft. Washington, PA, USA) during study involvement
5. Willing to return for all study visits and complete all study related procedures, including fasting before and during the hydrogen breath tests (HBTs)
6. Qualifying Lactose Challenge Symptom Score. Four symptom categories with severity measured from 0–5, as defined by one of the following:
  - At least one score of “moderately severe” or “severe” on a single symptom during the 6 h HBT
  - A score of “moderate” or greater for a single symptom at least two timepoints during the 6 h HBT
  - At least one “moderate” score or greater for each of two symptoms during the 6 h HBT
7. Able to understand and provide written informed consent in English.

Exclusion criteria:

1. Allergic to milk
2. Currently pregnant
3. Currently lactating
4. Cigarette smoking, or other use of tobacco or nicotine-containing products within 3 mo of screening
5. Diagnosed with any of the following disorders known to be associated with abnormal gastrointestinal (GI) motility: gastroparesis, amyloidosis, neuromuscular diseases (including Parkinson’s disease), collagen vascular diseases, alcoholism, uremia, malnutrition, or untreated hypothyroidism
6. History of surgery that alters normal GI tract function, including but not limited to: GI bypass surgery, bariatric surgery, gastric banding, vagotomy, fundoplication, pyloroplasty (N.B. history of uncomplicated abdominal surgeries such as removal of an appendix >12 months prior to screening will not be excluded)
7. Past or present: organ transplant, chronic pancreatitis, pancreatic insufficiency, symptomatic biliary disease, celiac disease, chronic constipation, diverticulosis, inflammatory bowel disease, ulcerative colitis, Crohn’s disease, small intestine bacterial overgrowth syndrome, gastroparesis, gastro-esophageal reflux disease, irritable bowel syndrome, or any other medical condition with symptoms that could confound collection of adverse events
8. Active ulcers, or history of severe ulcers
9. Diabetes mellitus (type 1 and type 2)
10. Congestive heart failure
11. Human immunodeficiency virus, hepatitis B, or hepatitis C
12. Body mass index > 35 kg/m^2^
13. Recent bowel preparation for endoscopic or radiologic investigation within 4 weeks of screening (e.g., colonoscopy preparation)
14. Use of concurrent therapy(ies) or other products (e.g., laxatives, stool softeners, Pepto Bismol^®^, Lactaid^®^ dietary supplements) used for symptoms of dairy intolerance within 7 days of screening
15. Chronic antacid and/or proton pump inhibitor use
16. Recent use of systemic antibiotics, defined as use within 30 days prior to screening
17. Recent high colonic enema, defined as use within 30 days prior to screening
18. Any concurrent disease or symptoms that may interfere with assessment of the cardinal symptoms of dairy intolerance (i.e., gas, diarrhea, bloating, cramps, stomach pain)
19. History of ethanol (alcohol) and/or drug abuse in the past 12 months
20. Currently undergoing chemotherapy
21. Use of any investigational drug or participation in any investigational study within 30 days prior to screening
22. Prior enrollment in this study
23. Any other conditions/issues noted by the study staff and/or Principal Investigator that would impact participation and/or protocol compliance.
